# Antioxidant Activity of Pharmaceuticals: Predictive QSAR Modeling for Potential Therapeutic Strategy

**DOI:** 10.3390/ph15070791

**Published:** 2022-06-24

**Authors:** Mario-Livio Jeličić, Jelena Kovačić, Matija Cvetnić, Ana Mornar, Daniela Amidžić Klarić

**Affiliations:** 1 Department of Pharmaceutical Analysis, Faculty of Pharmacy and Biochemistry, University of Zagreb, Ante Kovačića 1, 10000 Zagreb, Croatia; mljelicic@pharma.hr (M.-L.J.); jkovacic@pharma.hr (J.K.); amornar@pharma.hr (A.M.); 2 Department of Analytical Chemistry, Faculty of Chemical Engineering and Technology, University of Zagreb, Marulićev trg 19, 10000 Zagreb, Croatia

**Keywords:** pharmaceuticals, antioxidative activity, DPPH, HPLC, QSAR prediction

## Abstract

Since oxidative stress has been linked to several pathological conditions and diseases, drugs with additional antioxidant activity can be beneficial in the treatment of these diseases. Therefore, this study takes a new look at the antioxidant activity of frequently prescribed drugs using the HPLC-DPPH method. The antioxidative activity expressed as the TEAC value of 82 drugs was successfully determined and is discussed in this work. Using the obtained values, the QSAR model was developed to predict the TEAC based on the selected molecular descriptors. The results of QSAR modeling showed that four- and seven-variable models had the best potential for TEAC prediction. Looking at the statistical parameters of each model, the four-variable model was superior to seven-variable. The final model showed good predicting power (*r* = 0.927) considering the selected descriptors, implying that it can be used as a fast and economically acceptable evaluation of antioxidative activity. The advantage of such model is its ability to predict the antioxidative activity of a drug regardless of its structural diversity or therapeutic classification.

## 1. Introduction

Oxidative stress can be defined as an imbalance between the production of reactive oxygen radicals and, on the other side, the cell’s antioxidant capacity and exogenous antioxidant intake. Free radicals are generated from endogenous and exogenous sources and can cause significant chain chemical reactions in the body due to a quick reaction with other molecules. Oxidative stress has been linked to several pathological conditions and diseases, such as atherosclerosis and cardiovascular disease, cancer, neurological diseases (i.e., Alzheimer’s disease, amyotrophic lateral sclerosis, Parkinson’s disease, multiple sclerosis, depression, and memory loss), rheumatoid arthritis and respiratory diseases (asthma and chronic obstructive pulmonary disease). The basis of effective pharmacological treatment relies on both understanding the disease pathogenesis and the pharmacological drug effects. Although drugs belonging to the same therapeutic group have a common primary mechanism of action, pharmacokinetic and pharmacodynamic properties, as well as additional mechanisms of action, may differ among them. These diversities can single out one or more drugs as drugs of choice compared to other group members, especially in patients with comorbidities. According to the available literature data, antioxidant activity is especially underlined as one of the additional mechanisms of action that some drugs showed [1,2].

Quantitative structure–activity relation (QSAR) is a powerful tool for high-throughput virtual screening of various molecules based on the set of calculated descriptors representing each molecule [3]. Through the years, QSAR had a remarkable role in accelerating the drug development process. It was implemented in the various stages of this complex process, from the initial stages of drug design to pharmacokinetic and pharmacodynamic modeling [4,5,6]. The combination of a fast DPPH (2,2-diphenyl-1-picrylhydrazyl)-based method for antioxidant activity determination and QSAR is set to become a creating model for the prediction of the antioxidative activities of various compounds [7,8,9,10,11].

Since it is well recognized that reactive oxygen species are responsible for numerous types of cell damage, the objective of this study was to evaluate the antioxidant activity in vitro of frequently prescribed drugs in clinical practice. Accordingly, the antioxidative activity of 82 prescribing therapeutic agents from different Anatomical Therapeutic Chemical Classification (ATC) groups, such as antibiotics, antihistamines, beta-blockers, immunosuppressants and others, was determined using our previously developed DPPH-HPLC method [12]. Another objective was to develop a model for predicting antioxidant activity as an additional mechanism of action. Considering the rather large pool of structurally various drugs used in this high-throughput screening, obtained TEAC (TROLOX (6-hydroxy-2,5,7,8-tetramethylchroman-2-carboxylic acid) equivalent antioxidant capacity) values were used for QSAR model development to establish the unique model for the prediction of TEAC values. It must be pointed out that the advantage of such model is its ability to predict the antioxidative activity of a drug regardless of its structural diversity and therapeutic classification.

## 2. Results and Discussion

### 2.1. Method Verification

For DPPH assay monitoring, the applicability of our previously published method had to be verified following method transfer requirements. The method showed satisfactory linearity in the range from 0 to 0.3 mM of TROLOX (at seven concentration levels) with the following equation, *y* = −3.0101 *x* + 0.9997, and correlation coefficient, *r* = 0.9998. The accuracy of the method was examined by analyzing samples in triplicate on three different concentration levels, covering the whole range of the calibration curve, with the recoveries of 96.9% up to 104.1% accompanied by relative standard deviation values (RSD) no higher than 1.9%. The precision of the method was also examined by analyzing six samples at 0.15 mM of TROLOX with the RSD values not exceeding 5.8%.

### 2.2. The Antioxidative Activity of Selected Pharmaceuticals

The antioxidant activity of 82 pharmaceuticals was assessed by the HPLC-DPPH method (Table 1). According to obtained result, all analyzed drugs can be divided into three groups: (i) below 0.100 mM TEAC (63 pharmaceuticals; 77% of all), (ii) 0.100 to 0.200 mM TEAC (eight pharmaceuticals; 10% of all), and (iii) above 0.200 mM TEAC (11 pharmaceuticals; 13% of all).

The significant DPPH free-radical scavenging activity (0.059–0.141 mM TEAC) observed for all investigated statin (ATC class C10) drugs (atorvastatin, fluvastatin, pravastatin and simvastatin) bears a close resemblance to the one presented by previous research [13,14], although these authors used other experimental approaches. Our findings confirm that next to their already well-recognized antihyperlipemic and immunomodulatory effects, statins have a positive impact against oxidative stress levels.

Antibiotics (ATC class J) belong to one of the largest and structurally diverse group of pharmaceuticals. This study systematically improved our knowledge on the antioxidative activity of these widely prescribed medicines. Obtained values were in the range from 0.037 mM (azithromycin) to 0.302 mM (doxycycline) TEAC. Furthermore, the reaction of DPPH radicals and investigational antibiotics had not shown complete discoloration of the solution, as is the case with amoxicillin, erythromycin, sulfamethoxazole and sulphamic acid, but the applied HPLC method was sensitive enough to indicate small changes compared to the standard. It is interesting to note that cefradine, cephalexin and ciprofloxacin showed noticeable antioxidant activity, while oxytetracycline, doxycycline and rifampicin showed complete decolorization of DPPH solution, and have strong antioxidant activity. Despite antibiotics being frequently used pharmaceuticals, only a few researchers have addressed the question of their antioxidative activity. Kladna et al. [2] examined the antiradical activity of tetracyclines, including oxytetracycline and doxycycline, using DPPH, while Karunakar et al. [1] used the HPLC-DPPH method to determine the antioxidant activity of drugs, including rifampicin. The results showed that in the presence of rifampicin, the concentration of DPPH peaks decreased depending on the concentration. Furthermore, the free radical scavenging activity of rifampicin is measured by Kalpana et al. [15] and ascorbic acid was used for comparison. Although the above-mentioned researchers used different procedures, the results of this work are consistent with the available literature.

Analgesics (ATC class N02) are one of the most commonly self-prescribed medicines. The benefit of the combined use of analgesics and antioxidants in the treatment of chronic pain is attracting considerable interest [16,17]. It is well known that paracetamol is often used in combination with vitamin C. In this study, the ability to capture the DPPH radical of the above analgesic was assessed (0.236 mM TEAC). Borges et al. [18] evaluated the antioxidant activity of paracetamol and salicylic acid in experimental and theoretical studies. In conclusion, it is stated that although both compounds are phenolic derivatives, paracetamol showed more pronounced antioxidant properties than salicylic acid in several models that caused oxidative stress. One of the theoretical mechanisms has shown that hydrogen transfer is responsible for a more pronounced antioxidant effect in paracetamol. On the other hand, there are numerous examples in the available literature of the toxicity of paracetamol, which is widely used as an analgesic and antipyretic. Although seemingly safe if used at the recommended therapeutic doses, higher doses of paracetamol can cause severe liver and kidney damage in humans and experimental animals.

Based on the obtained results (Table 1), aminosalicylates (ATC class A07) stand out as an interesting group of investigated drugs. Sulfasalazine (0.076 mM TEAC) and mesalazine (5-ASA; 0.296 mM TEAC) showed measurable antioxidant activities, while the antioxidant activities of olsalazine and balsalazide were not measurable by the proposed HPLC-DPPH method. Rafael et al. [19] used the DPPH radical in the analysis of mesalazine to quantify the content of the drug in finished, commercially available pharmaceutical forms using the spectrophotometric method. Additionally, a literature survey reveals that mesalazine had a potent radical scavenger activity compared to paracetamol and salicylic acid, as antipyretic and anti-inflammatory drugs [20]. Interestingly, the intensity of the antioxidant effect of mesalazine was similar to ascorbate, in contrast to salicylate, which did not react with the DPPH radical. Furthermore, these results suggest that most of the antioxidant activity of aminosalicylates is derived from 5-ASA. By being released from the prodrug structure due to the enzymatic degradation of the azo bond, the antioxidant activity could be part of the therapeutic effect of sulfasalazine, olsalazine and balsalazide, even if their prodrug activity was much weaker than 5-ASA alone. The highest antioxidative activity was found for 0.1 mM mesalazine (up to 310 times stronger than others), followed by aminosalicylates, sulfasalazine and balsalazide. On the other hand, olsalazine has shown no antioxidant activity.

According to the results of this study, the antioxidative activity of immunosuppressant drugs (ATC class L04) was observed: 6-mercaptopurine and 6-thioguanine showed twice as much antioxidative power compared to prodrug azathioprine.

As already mentioned, oxidative stress plays an important role in the degeneration of dopaminergic neurons in Parkinson’s disease. Ropinirole is a non-ergoline D2/D3 dopamine agonist (ATC class N04) used to treat symptoms of Parkinson’s disease and showed increasing free radical scavenging activity with increasing concentrations. Our results share a number of similarities with Selva et al.’s findings, as this group showed that ropinirole had good free radical scavenging activity and could play a role of a neoadjuvant antioxidant in a wide variety of neurodegenerative disorders [21].

Vitamins have been recognized as one of the essential antioxidants. Folic acid (vitamin B9) had significant antioxidant activity (0.230 mM TEAC), and other B vitamins, such as thiamine (vitamin B1) and nicotinamide, a form of vitamin B3, showed measurable antioxidant activity (0.115 mM TEAC and 0.048 mM TEAC, respectively). The obtained results extended our knowledge of them and demonstrated the highest antioxidant activity of L-ascorbic acid sodium salt (0.267 mM TEAC) compared to other investigated vitamins.

### 2.3. Development of QSAR Model for the Prediction of Antioxidative Activity of Drugs

The following uses the procedure described in Section 3.3. In the statistical procedure, we approached the development of the QSAR model using the obtained TEAC values and calculated descriptors. Data splitting to create training and the test set was performed using the rational splitting method based on activity value, to cover the whole range of TEAC values [22]. As a result, one- to eight-variable models were obtained. Equations of the developed models are presented in Table 2.

The values of the statistical parameters of selected one-, two-, three-, four-, five-, six-, seven- and eight-variable QSAR models for the training and the test set are presented in Table 3. To satisfy the fitting criteria of the model, parameters should act as follows: *R*^2^ values should tend to be as high as 1, implying that calculated values are similar to the observed ones. The acceptable *R*^2^ value of a model should be ≥0.6 (equivalent to *r* ≥ 0.774). The minimum acceptable *R*^2^_ext_ is ≥0.6 as well; *CCC* ≥ 0.85; *RMSE* and *MAE* as close to zero as possible and *RMSE_tr_* should be of smaller value than RMSE_cv_. Robust QSAR models should have *R*^2^ yscr > *Q*^2^ yscr [23].

**Figure 1:**
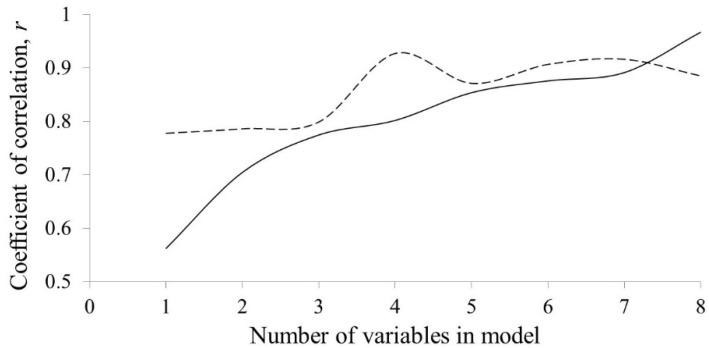
Plot showing the obtained *r* values in relation to variables in the model for the training set (full line) and test set (dashed line).

In addition, plots of obtained correlation coefficients, *r*, in relation to the number of variables in the obtained *QSAR* models are shown in Figure 1.

The *r* values for the training set models increase with the increment of variable number in the model (from 0.562 for the one-variable model, up to 0.914 for the eight-variable model); however, *r* values of obtained test set models have shown that four- and seven- variable models have highest values (0.927 and 0.916, respectively) compared to other models (from 0.778 to 0.907). Although the seven-variable model exhibits a higher *r* value of the training set (0.891) and a somewhat similar *r* value for the test set, the four-variable model showed better predictive power. Both have a similar performance of external and internal validation parameters; however, a crucial part in deciding which model is more suitable for the prediction of TEAC played was the cross-correlation matrix of the seven-variable model. Table 4 shows that several descriptors are highly correlated (H7s vs. CATS2D_04_AL, *r* = 0.759 and C-018 vs. F03[N-F], *r* = 0.766), meaning that both descriptors have the same contribution to the model outcome, thus one of them is unnecessary in the model, which is not the case in the four-variable model, in which the highest correlation of descriptors is 0.586, implying that every descriptor contributes to calculation of the TEAC value. On the other hand, generating models with more than eight variables would increase *r* of the training set; however, in the case of test sets, lower *r* values would be obtained, as is the case in the eight-variable model, due to the overtraining of the model. Therefore, building higher models with more than eight variables would not be efficient. The list of all descriptors included in one- to eight- variable models are presented and will be described in detail in the following section.

**Figure 2:**
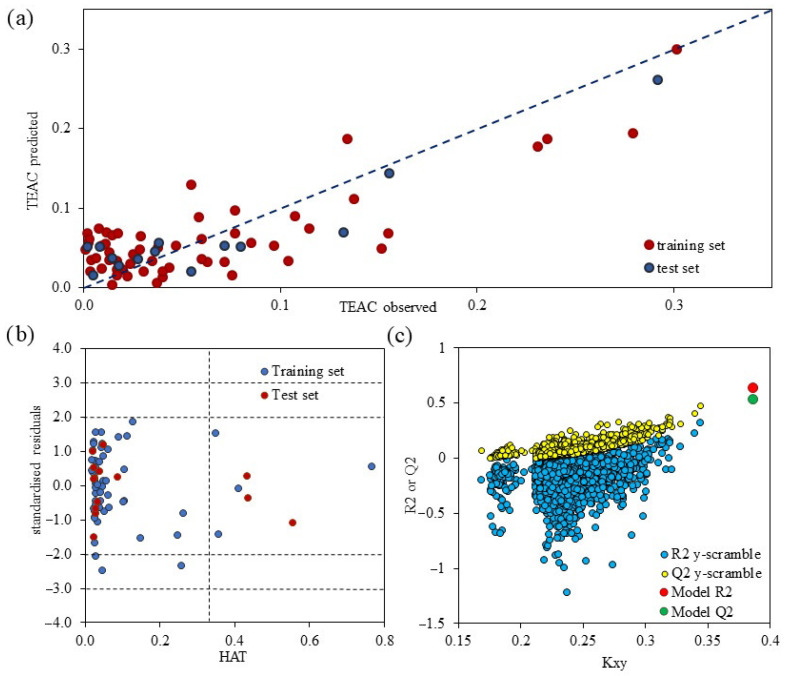
Plots representing (**a**) model linearity, (**b**) Williams plot and (**c**) validation using “Y-scrambling”.

Based on the facts stated above, the four-variable model was chosen as the best one for the prediction of TEAC. Model accuracy, as well as the domain of applicability, is presented in Figure 2. Figure 2A shows a plot of predicted TEAC values versus the measured ones, resulting in a distribution of values that show a linear trend. The points are distributed along the diagonal line, representing the accuracy of the model as well as the fact that the model was able to predict similar TEAC values, especially for the molecules that have shown strong antioxidative power. One cluster comprised molecules that exhibit low antioxidative power. On the other hand, the Williams plot detects both the response outliers (Y-outliers) and molecular structure outliers (X-outliers) [24]. As can be seen in Figure 2B, there are no response outliers since none of the values exceed ± 3*δ*. On the other hand, structurally influential molecules were observed (three in the test set and four in the training set), which is acceptable considering the structural diversity in the pool of examined molecules.

The model was further validated using the “Y-scrambling” method (Figure 2C). The “Y-scrambling” test showed that *R*^2^ and *Q*^2^_LOO_ values of selected models are different than those calculated. The observed *R*^2^_Y-SCRAMBLING_ and *Q*^2^_Y-SCRAMBLING_ are of low value, indicating the validity of the calculated model. Additionally, the *R*^2^_Y-SCRAMBLING_ > *Q*^2^_Y-SCRAMBLING_ criteria are satisfied.

### 2.4. Structural Characteristics Determining the Antioxidative Value of Selected Compounds

The list of descriptors in the one- to eight-variable models is presented in Table 5. In this section, the focus will be primarily on the descriptors found in the four-variable model, which was chosen as the best one for the prediction of TEAC.

Considering the observed equation describing the four-variable model:

*TEAC* = −0.0851(±0.0533) × *Mor16e* − 0.1511(±0.0964) × *RDF145p* + 0.1489(±0.0526) × *C-018* + 0.1991(±0.0694) × *CATS2D_06_AL* + 0.0396(±0.0156)

descriptors of interest are the following: Mor16e, RDF145p, C-018 and CATS2D_06_AL, belonging to the 3D-MoRSE, Radial Distribution Function, =CHX and CATS 2D type of descriptors, respectively (Table 5).

The first descriptor found in our four-variable model is the Mor16e descriptor, belonging to the group of 3D-MoRSE descriptors, which stands for 3D-Molecule Representation of Structures based on electron diffraction. These descriptors were developed based on the idea of obtaining information from the 3D atomic coordinates by use of the transform used in electron diffraction studies for preparing theoretical scattering curves. The MoRSE descriptors have been shown to have good modeling power for different biological and physicochemical properties and can be used even for the simulation of infrared spectra [25]. In our model, we can see the negative contribution of the Mor16e descriptor, which represents signal16/weighted by Sanderson electronegativity. According to Sanderson’s theory, a compound that exhibits high electronegativity, due to the equalization of electronegativity between two atoms, is associated with low reactivity; therefore, the lower the Mor16e value associated with the compound, the more pronounced its antioxidative potential is [26]. 3D-MoRSE type descriptors were also part of models obtained in the previously published studies for the prediction of antioxidative activity [23].

The second descriptor found in the obtained model is RDF145p. Radial Distribution Function (RDF) descriptors are based on the geometrical interatomic distance and constitute a radial distribution function code, and they show some characteristics in common with the 3D-MoRSe descriptors [25]. They contain information about the interatomic distances in a molecule, unweighted or weighted by different atomic properties, such as atomic mass, electronegativity, van der Waals volume and atomic polarizability [27]. We can see that increase in those properties of the molecule can negatively affect the antioxidative power of a compound due to the negative value in the obtained model.

The third descriptor in our model, C-018, belongs to the atom-centered fragment type of descriptors. Each atom type is an atom in the molecule described by its neighboring atoms. Hydrogen and halogen atoms are classified by the hybridization and oxidation state of the carbon atom to which they are bonded. Carbon atoms are classified by their hybridization state and depending on whether their neighbors are carbon or heteroatoms. C-018 is defined as =CHX atom-centered fragment, where X can represent any electronegative atom (O, N, S, P, Se, halogens) as well as an aromatic bond, as in benzene [28]. This descriptor strongly contributes to the TEAC values calculated by our model.

The last descriptor found in our model is from the group of CATS 2D descriptors, named CATS_2D_AL. The CATS 2D (Chemically Advanced Template Search) descriptors are a type of autocorrelation descriptors, where the atom-type definition is related to potential pharmacophore points (PPP). CATS2D descriptors are widely used for similarity search 17. The names of the CATS2D descriptors are coded as follows: “CATS2D_”, “distance2D_”, and “type atom pair”. Thus, “CATS2D_06_AL” means the count of all molecular graph distances (6) between atom pairs are acceptor lipophilic (AL) [29]. It is visible that this descriptor has a positive impact on predicted TEAC values.

## 3. Materials and Methods

### 3.1. Chemicals and Reagents

Standards of pharmaceuticals selected for this study (Table 1) were obtained by the following manufacturers: Sigma-Aldrich (St. Louis, MO, USA), Fluka (Buchs, Switzerland), TCI (Tokyo, Japan), Ph. Eur. 7.0 and Pliva d.o.o. (Zagreb, Croatia). DPPH (95%), a free radical, and TROLOX (6-hydroxy-2,5,7,8-tetramethylchroman-2-carboxylic acid), a synthetic antioxidant and water-soluble analog of vitamin E, were purchased from Sigma-Aldrich (St. Louis, MO, USA). Methanol (98–100%; HPLC grade) was delivered by Merck (Darmstadt, Germany). Ultra-pure water from a Mili-Q water purification system (Millipore, St. Louis, MO, USA) with a resistivity of 18.2 MΩ cm (25 °C) was used.

### 3.2. HPLC-DPPH Method

The antioxidative activity of selected drugs was determined using the DPPH method followed by chromatographic analysis. Briefly, after 250 µL of 2.5 mM DPPH methanolic solution was added to 1 mL of 1 mM drug solution, the reaction mixture was stirred and stored in the dark for 30 min at room temperature. A chromatographic analysis was conducted on Agilent 1100 HPLC system (Agilent Technologies, Santa Clara, CA, USA) with diode array detector using our previously published method adopted for these specific samples [12]. For this reason, method verification was performed to confirm the applicability of the method to the different chromatographic systems. Determination of the antioxidant activity of the drug was performed in triplicate.

The ability of each drug sample to scavenge the ‘stable’ free DPPH radical was determined from the difference in the peak area of the initial solution of the radical itself and the solution of the radical after reaction with the sample. TROLOX was used as a standard antioxidant and the results were expressed as TEAC values determined from a standard calibration curve.

### 3.3. Statistical Procedure

#### 3.3.1. Data Set and Calculation of Descriptors

A set of 82 pharmaceuticals with accompanying TEAC values was used in the QSAR study. The set was divided into the training set, comprising 68 structurally various drugs, and the prediction set, which also included 14 structurally various drugs.

For calculation of descriptors, 3D structures of selected compounds were created in Chem3D Pro software (ChemOffice v15.0, Perkin Elmer, Waltham, MA, USA). Molecular conformations were optimised by the AM1 method using the MOPAC2012 interface. A total of 3242 molecular descriptors were calculated using the DRAGON v6.0 software (Milano chemometrics & QSAR research group, Milano, Italy), describing the chemical diversity of the studied set as well as capturing the relevant structural characteristics.

#### 3.3.2. Statistical Correlation

To calculate the correlation between TEAC (response values used in QSAR) and DRAGON-generated structurally related descriptors, Genetic Algorithm (GA) and Multiple Linear Regression Analysis (MLRA) methods were used. The combination of the GA-MLRA methods was applied for the selection of descriptors and construction of one-, two-, three-, four-, five-, six-, seven- and eight-variable models using QSARINS v2.2. (QSAR Group, University of Insubria, Varese, Italy). GA variable selection technique started with a population of 200 random models and 2000 iterations to the evolution, with the mutation probability specified as 20%. All descriptors used in calculations of models were expressed as their normalized values for easier comparison of their contribution to QSAR responses. To exclude the models with highly cross-correlated descriptors as well as models with low correlation, filtering through the QUIK rule built-in QSARINS software was performed. Additionally, models with over-correlated descriptors as well as non-significant correlation coefficients were excluded from further study.

The best models were selected according to their correlation coefficients (*r*), model variance (*R*^2^), F-ratio, leave-one-out cross-validation (*Q*^2^), standard error (*s*) and standard error of the predictive residue of sum of squares (S_PRESS_). The validation of the model was performed using leave-many-out (LMO) and “Y-scrambling” tests. A Williams plot was used to visualize the applicability domain (AD) of developed models. Such a plot of standardized cross-validated residuals (RES) vs. leverage (Hat diagonal) values (HAT) depicts both the response outliers (Y outliers) and structurally influential compounds (X outliers) in a model.

## 4. Conclusions

In this study, the applied HPLC-DPPH method has proven to be rapid and effective for determining the antioxidant activity of eighty-two pharmaceuticals in order to investigate additional mechanisms of their action that are widely used in clinical practice. In total, 23% of the analyzed pharmaceuticals had good free radical scavenging activity (above 0.100 mM TEAC) and could play the role of a neoadjuvant antioxidant.

Results of QSAR modelling showed that four- and seven-variable models had the best potential for prediction of TEAC. Looking into statistical parameters of each model, four-variable model showed to be superior to seven-variable model, due to the fact that seven-variable model showed highly cross-correlated descriptors, meaning that there is a lack of information variability. The chosen model showed good linearity and was successfully validated and tested. With the correlation coefficient of 0.927 for predicted TEAC values, it is shown that this model is adequate for the prediction of TEAC values considering the descriptors included in the model.

The conducted study systematically improved the knowledge of the antioxidative activity of these widely prescribed medicines and provide a scientific basis for the subsequent elucidation of the pharmaceuticals and their additional mechanism activities. Overall, the results lay the foundation for in-depth research on the antioxidative activity of drugs with high TEAC values. Furthermore, this research strategy can be used for the drug characterization according to various antioxidant tests and for enabling the further development and utilization of these pharmaceuticals.

## Figures and Tables

**Table 1:** Description of investigated pharmaceuticals.

Name	CAS	Molecular Formula	Pharmacologic Class	ATC *	TEAC (mM)
Acetazolamide	59-66-5	C_4_H_6_N_4_O_3_S_2_	Mitotic	S	0.009
Amoxicillin	26787-78-0	C_16_H_19_N_3_O_5_S	Antibiotic	J	0.085
Antipyrine (Phenazone)	60-80-0	C_11_H_12_N_2_O	Analgetic	N	0.013
Atenolol	29122-68-7	C_14_H_22_N_2_O_3_	Beta blocker	C	0.037
Atorvastatin	134523-00-5	C_33_H_35_FN_2_O_5_	Hypolipemic	C	0.059
Atropine sulphate	5908-99-6	C_34_H_50_N_2_O_11_S	Spasmolytic	A, S	0.010
Azathioprine	446-86-6	C_9_H_7_N_7_O_2_S	Immunosuppressive	L	0.001
Azithromycin	83905-01-5	C_38_H_72_N_2_O_12_	Antibiotic	J	0.037
Balsalazide	80573-04-2	C_17_H_15_N_3_O_6_	Aminosalicylate	A	0.008
Barbital	57-44-3	C_8_H_12_N_2_O_3_	Sedative	N	0.001
Benzocaine (4-aminobenzoate)	94-09-7	C9H11NO2	Anaesthetic	N	0.004
Bisoprolol	66722-44-9	C_18_H_31_NO_4_	Beta blocker	C	0.008
Caffeine	58-08-2	C_8_H_10_N_4_O_2_	Analeptic	N	0.013
Carvedilol	72956-09-3	C_24_H_26_N_2_O_4_	Beta blocker	C	0.028
Cefalexin	15686-71-2	C_16_H_17_N_3_O_4_S	Antibiotic	J	0.170
Cefradine	38821-53-3	C_16_H_19_N_3_O_4_S	Antibiotic	J	0.152
Chloramphenicol	56-75-7	C_11_H_12_Cl_2_N_2_O_5_	Antibiotic	J	0.041
Cimetidine	51481-61-9	C_10_H_16_N_6_S	H2-receptor antagonist	A	0.018
Ciprofloxacin	85721-33-1	C_17_H_18_FN_3_O_3_	Antibiotic	J	0.155
Clarithromycin	81103-11-9	C_38_H_69_NO_13_	Antibiotic	J	0.044
Codeine phosphate	41444-62-6	C_18_H_24_NO_7_P	Analgetic	N	0.017
Diazepam	439-14-5	C_16_H_13_ClN_2_O	Sedative	N	0.00001
Digoxin	20830-75-5	C_41_H_64_O_14_	Cardiotonic	C	0.00001
Docetaxel	148408-66-6	C_43_H_59_NO_17_	Cytostatic	L	0.001
Doxycycline	564-25-0	C_22_H_24_N_2_O_8_	Antibiotic	J	0.302
Dopamine	62-31-7	C_8_H_12_ClNO_2_	Dopamine	C	0.274
Ephedrine	299-42-3	C_10_H_15_NO	Adrenergic	C	0.002
Erythromycin	114-07-8	C_37_H_67_NO_13_	Antibiotic	J	0.080
Febuxostat	144060-53-7	C_16_H_16_N_2_O_3_S	Non-purine xanthine oxidase inhibitor	M	0.00001
Fluvastatin	93957-55-2	C_24_H_25_FNNaO_4_	Hypolipemic	C	0.138
Folic acid	59-30-3	C_19_H_19_N_7_O_6_	Vitamin	A	0.230
Furosemide	54-31-9	C_12_H_11_ClN_2_O_5_S	Diuretic	C	0.055
Gemcitabine	122111-03-9	C_9_H_12_ClF_2_N_3_O_4_	Cytostatic	L	0.060
Hydrochlorothiazide	58-93-5	C_7_H_8_ClN_3_O_4_S_2_	Diuretic	C	0.018
Ibuprofen	15687-27-1	C_13_H_18_O_2_	NSAID **	M	0.005
Ketoprofen	22071-15-4	C_16_H_14_O_3_	NSAID	M	0.006
L-Ascorbic acid sodium salt	134-03-2	C_6_H_8_O_6_	Vitamin	A	0.267
6-Mercaptopurine	50-44-2	C_5_H_4_N_4_S	Immunosuppressive	L	0.292
Mesalazine	89-57-6	C_7_H_7_NO_3_	Aminosalicylate	A	0.296
Metronidazole	443-48-1	C_6_H_9_N_3_O_3_	Antibiotic	J	0.015
Nebivolol	99200-09-6	C_22_H_25_F_2_NO_4_	Beta blocker	C	0.017
Nifedipine	21829-25-4	C_17_H_18_N_2_O_6_	Calcium channel blocker	C	0.029
Nicotinamide	98-92-0	C_6_H_6_N_2_O	Vitamin	A	0.048
O-Acetylsalicylic acid	50-78-2	C_9_H_8_O_4_	NSAID	B, N	0.012
Oxazepam	604-75-1	C_15_H_11_N_2_O_2_Cl	Sedative	M	0.007
Oxytetracycline	79-57-2	C_22_H_24_N_2_O_9_	Antibiotic	J	0.299
Olsalazine	6054-98-4	C_14_H_8_N_2_Na_2_O_6_	Aminosalicylate	A	0.002
Pantoprazole	102625-70-7	C_16_H_14_F_2_N_3_NaO_4_S	Proton-pump inhibitor	A	0.030
Calcium pantothenate	443753	C_18_H_32_CaN_2_O_10_	Vitamin	A	0.002
Papaverine	61-25-6	C_20_H_22_ClNO_4_	Spasmolytic	A	0.012
Paracetamol	103-90-2	C_8_H_9_NO_2_	Analgetic	N	0.236
Phenobarbitone	50-06-6	C_12_H_12_N_2_O_3_	Sedative	N	0.038
Physostigmine salicylate	57-64-7	C_22_H_27_N_3_O_5_	Parasympathomimetic	S	0.063
Piperazine	142-63-2	C_4_H_22_N_2_O_6_	Anthelmintic	P	0.132
Piracetam	7491-74-9	C_6_H_10_N_2_O_2_	Antidepressant	N	0.025
Pirfenidone	53179-13-8	C_12_H_11_NO	Anti-inflammatory, antifibrotic	L	0.00001
Pravastatin sodium	81131-70-6	C_23_H_35_NaO_7_	Hypolipemic	C	0.097
Procaine	51-05-8	C_13_H_21_ClN_2_O_2_	Anaesthetic	N	0.024
Propyphenazone	479-92-5	C_14_H_18_N_2_O	Analgetic	N	0.003
Propranolol	525-66-6	C_16_H_21_NO_2_	Beta blocker	C	0.00001
Quetiapine fumarate	111974-72-2	C_46_H_54_N_6_O_8_S	Antipsychotic	N	0.030
Quinidine	56-54-2	C_20_H_24_N_2_O_2_	Antiarrhythmic agent	C	0.029
Quinin sulphate	207671-44-1	C_40_H_50_N_4_O_8_S	Antimalaria	P	0.017
Rifampicin	13292-46-1	C_43_H_58_N_4_O_12_	Antibiotic	J	0.292
Risperidone	106266-06-2	C_23_H_27_FN_4_O_2_	Antipsychotic	N	0.010
Ropinirole	91374-20-8	C_16_H_25_ClN_2_O	Anti-Parkinson’s drug	N	0.285
Salicylic acid	69-72-7	C_7_H_6_O_3_	Anti-inflammatory, antibacterial	D	0.024
Sildenafil citrate	171599-83-0	C_22_H_30_N_6_O_4_S	PDE 5 inhibitor	G	0.003
Simvastatin	79902-63-9	C_25_H_38_O_5_	Hypolipemic	C	0.141
Sulfacetamide sodium	6209-17-2	C_8_H_11_N_2_NaO_4_S	Antibiotic	J	0.015
Sulfadiazine	68-35-9	C_10_H_10_N_4_O_2_S	Antibiotic	J	0.015
Sulfamethoxazole	723-46-6	C_10_H_11_N_3_O_3_S	Antibiotic	J	0.077
Sulfasalazine	599-79-1	C_18_H_14_N_4_O_5_S	Aminosalicylate	A	0.076
Sulfathiazole	72-14-0	C_9_H_9_N_3_O_2_S_2_	Antibiotic	J	0.035
Sulphamic acid	5329-14-6	NH_2_SO_3_H	Antibiotic	J	0.104
Sulphanilamide	63-74-1	C_6_H_8_N_2_O_2_S	Antibiotic	J	0.023
6-Thioguanine	154-42-7	C_5_H_5_N_5_S	Immunosuppressive	L	0.288
Theobromine	83-67-0	C_7_H_8_N_4_O_2_	Antiasthmatic	R	0.011
Theophylline	58-55-9	C_7_H_8_N_4_O_2_	Antiasthmatic	R	0.004
Thiamine	67-03-8	C_12_H_18_Cl_2_N_4_OS	Vitamin	A	0.115
Warfarin	81-81-2	C_19_H_16_O_4_	Anticoagulant	B, N	0.077
Zopiclone	43200-80-2	C_17_H_17_ClN_6_O_3_	Sedative	N	0.018

* ATC—Anatomical Therapeutic Chemical Clasification. ** NSAID—Non-steroidal anti-inflammatory drugs.

**Table 2:** Selected 1- to 8-variable models for prediction of TEAC on the training set.

Variable No.		Equation
1	*TEAC*	= 0.1739(±0.0687) × C-018 + 0.0500(±0.0156)
2	*TEAC*	= 0.1760(±0.0595) × C-018 + 0.1487(±0.0673) × H7s + 0.0287(±0.0166)
3	*TEAC*	= 0.1835(±0.0529) × C-018 + 0.1464(±0.0580) × CATS2D_06_AL − 0.1050(±0.0487) × Mor24e + 0.0458(±0.0157)
4	*TEAC*	= −0.0851(±0.0533) × Mor16e − 0.1511(±0.0964) × RDF145p + 0.1489(±0.0526) × C-018 + 0.1991(±0.0694) × CATS2D_06_AL + 0.0396(±0.0156)
5	*TEAC*	= −0.1657(±0.0819) × RCI − 0.1458(±0.0848) × RDF145p − 0.0847(±0.0469) Mor16e + 0.1484(±0.0463) × C-018 + 0.2113(±0.0613) × CATS2D_06_AL + 0.1292(±0.0463)
6	*TEAC*	= −0.1686(±0.0768) × RCI − 0.1695(±0.0811) × RDF145p − 0.0801(±0.0456) × Mor16e + 0.1388(±0.0788) × H7s + 0.1579(±0.0435) × C-018 + 0.1061(±0.0712) × CATS2D_04_AL + 0.1175(±0.0439)
7	*TEAC*	= −0.1700(±0.0728) × RCI − 0.1717(±0.0764) × RDF145p − 0.0965(±0.0481) × Mor16u + 0.1412(±0.0745) × H7s + 0.2087(±0.0625) × C-018 + 0.1086(±0.0682) × CATS2D_04_AL − 0.1046(±0.0952) × F03[N-F] + 0.1194(±0.0416)
8	*TEAC*	= −0.1880(±0.0673) × RCI − 0.1821(±0.0703) × RDF145p − 0.0861(±0.0442) × Mor16u + 0.0811(±0.0753) × H7s + 0.0606(±0.0386) × nR = Ct + 0.1483(±0.0383) × C-018 + 0.1236(±0.0622) × CATS2D_04_AL + 0.0764(±0.0529) × F06[N-F] + 0.1241(±0.0384)

**Table 3:** Statistical parameters of obtained one- to eight-variable models.

Model No.	1	2	3	4	5	6	7	8
N_tr_	58	58	58	58	58	58	58	58
N_ex_	14	14	14	14	14	14	14	14
**Fitting Criteria**								
*R* ^2^	0.316	0.496	0.599	0.643	0.729	0.767	0.794	0.835
*R* ^2^_adj_	0.304	0.478	0.578	0.616	0.703	0.739	0.766	0.808
*s*	0.0576	0.0499	0.0499	0.0428	0.0377	0.0353	0.0334	0.0302
*F*	25.885	27.109	26.986	27.846	27.974	27.977	27.629	31.059
*p*	<0.0000	<0.0000	<0.0000	<0.0000	<0.0000	<0.0000	<0.0000	<0.0000
*K*xx	0.000	0.016	0.034	0.232	0.187	0.318	0.359	0.309
Δ*K*	0.562	0.337	0.238	0.154	0.135	0.073	0.053	0.060
RMSE_tr_	0.056	0.49	0.043	0.041	0.036	0.033	0.031	0.028
MAE_tr_	0.042	0.037	0.034	0.033	0.028	0.025	0.023	0.021
CCC_tr_	0.480	0.664	0.750	0.886	0.843	0.868	0.783	0.910
**Internal Validation Criteria**								
*Q* ^2^_LOO_	0.227	0.339	0.462	0.539	0.632	0.687	0.724	0.748
RMSE_cv_	0.060	0.056	0.050	0.047	0.041	0.038	0.036	0.034
MAE_cv_	0.044	0.041	0.038	0.037	0.032	0.030	0.028	0.026
PRESS_cv_	0.210	0.180	0.146	0.126	0.100	0.085	0.075	0.068
CCC_cv_	0.419	0.564	0.663	0.854	0.786	0.821	0.845	0.862
**External Validation Criteria**								
RMSE_ext_	0.049	0.052	0.052	0.030	0.043	0.034	0.031	0.042
MAE_ext_	0.040	0.043	0.041	0.026	0.033	0.029	0.024	0.033
PRESS_ext_	0.033	0.038	0.038	0.013	0.026	0.015	0.013	0.025
*R* ^2^_ext_	0.657	0.618	0.639	0.859	0.759	0.822	0.839	0.784
*Q* ^2^_F1_	0.606	0.544	0.551	0.845	0.687	0.809	0.840	0.707
*Q* ^2^_F2_	0.601	0.539	0.546	0.843	0.684	0.807	0.839	0.704
*Q* ^2^_F3_	0.498	0.419	0.429	0.803	0.601	0.756	0.797	0.627
CCC_ext_	0.703	0.784	0.792	0.913	0.777	0.883	0.907	0.805

**Table 4:** Correlation matrix of included descriptors in best four- and seven-variable QSAR models predicting TEAC for the entire set of compounds (72).

**4-Variable Model**							
	Mor16e		RDF145p		C-018		CATS2D_06_AL
Mor16e	1		0.154		0.247		0.005
RDF145p			1		0.058		0.586
C-018					1		0.011
CATS2D_06_AL							1
**7-Variable Model**							
	RCI	RDF145p	Mor16u	H7s	C-018	CATS2_04_AL	F03[N-F]
RCI	1	0.110	0.007	0.157	0.010	0.115	0.008
RDF145p		1	0.155	0.558	0.058	0.585	0.045
Mor16u			1	0.056	0.247	0.150	0.219
H7s				1	0.016	0.759 *	0.008
C-018					1	0.091	0.766 *
CATS2D_04_AL						1	0.092
F03[N-F]							1

* >cross-correlation *R*_ij_ = 0.7.

**Table 5:** Names and definitions of descriptors included in the best one–eight variable models for prediction of TEAC.

Descriptor Name	Model	Descriptor Definition	Descriptor Type
C-018	1-, 2-, 3-, 4-, 5-, 6-, 7- and 8-variable	=CHX	Atom-centred fragments
H7s	2-, 6-, 7- and 8-variable	H autocorrelation of lag 7/weighted by I-state	GETAWAY descriptors
CATS2D_06_AL	3-, 4- and 5-variable	CATS2D Acceptor-Lipophilic at lag 06	CATS 2D
Mor24e	3-variable	signal 24/weighted by Sanderson electronegativity	3D-MoRSE descriptors
Mor16e	4-, 5- and 6-variable	signal 16/weighted by Sanderson electronegativity	3D-MoRSE descriptors
RDF145p	4-, 5-, 6-, 7- and 8-variable	Radial Distribution Function—145/weighted by polarizability	RDF descriptors
RCI	5-, 6-, 7- and 8-variable	ring complexity index	Ring descriptors
CATS2D_04_AL	6-, 7- and 8-variable	CATS2D Acceptor-Lipophilic at lag 04	CATS 2D
Mor16u	7- and 8-variable	signal 16/unweighted	3D-MoRSE descriptors
F03[N-F]	7-variable	Frequency of N—F at topological distance 3	2D Atom Pairs
nR = Ct	8-variable	number of aliphatic tertiary C(sp2)	Functional group counts
F06[N-S]	8-variable	Frequency of N—S at topological distance 6	2D Atom Pairs

## Data Availability

Data is contained within the article.

## References

[1] Karunakar N., Prabhakar M.C., Krishna D.R. (2003). Determination of antioxidant activity of some drugs using high-pressure liquid chromatography. Arzneimittel-Forschung/Drug Res..

[2] Kładna A., Michalska T., Berczyński P., Kruk I., Aboul-Enein H.Y. (2012). Evaluation of the antioxidant activity of tetracycline antibiotics in vitro. Luminescence.

[3] Kwon S., Bae H., Jo J., Yoon S. (2019). Comprehensive ensemble in QSAR prediction for drug discovery. BMC Bioinform..

[4] Castillo-Garit J.A., Abad C., Rodríguez-Borges J.E., Marrero-Ponce Y., Torrens F. (2012). A review of QSAR studies to discover new drug-like compounds actives against leishmaniasis and trypanosomiasis. Curr. Top. Med. Chem..

[5] Neves B.J., Braga R.C., Melo-Filho C.C., Moreira-Filho J.T., Muratov E.N., Andrade C.H. (2018). QSAR-based virtual screening: Advances and applications in drug discovery. Front. Pharmacol..

[6] Vieira J.B., Braga F.S., Lobato C.C., Santos C.F., Costa J.S., Bittencourt J.A.H.M., Brasil D.S.B., Silva J.O., Hage-Melim L.I.S., Macêdo W.J.C. (2014). A QSAR, pharmacokinetic and toxicological study of new artemisinin compounds with anticancer activity. Molecules.

[7] Duchowicz P.R., Szewczuk N.A., Pomilio A.B. (2019). QSAR studies of the antioxidant activity of anthocyanins. J. Food Sci. Technol..

[8] Razo-Hernández R.S., Pineda-Urbina K., Velazco-Medel M.A., Villanueva-García M., Sumaya-Martínez M.T., Martínez-Martínez F.J., Gómez-Sandoval Z. (2014). QSAR study of the DPPH• radical scavenging activity of coumarin derivatives and xanthine oxidase inhibition by molecular docking. Cent. Eur. J. Chem..

[9] Abreu R.M.V., Ferreira I.C.F.R., Queiroz M.J.R.P. (2009). QSAR model for predicting radical scavenging activity of di (hetero) arylamines derivatives of benzo [b] thiophenes. Eur. J. Med. Chem..

[10] Tran T.T.N., Tran D.P., Nguyen V.C., Tran T.D.T., Bui T.T.T., Bowie J.H. (2021). Antioxidant activities of major tryptophyllin L peptides: A joint investigation of Gaussian-based 3D-QSAR and radical scavenging experiments. J. Pept. Sci..

[11] Martínez-Martínez F.J., Razo-Hernández R.S., Peraza-Campos A.L., Villanueva-García M., Sumaya-Martínez M.T., Cano D.J., Gómez-Sandoval Z. (2012). Synthesis and in vitro antioxidant activity evaluation of 3-carboxycoumarin derivatives and QSAR study of their DPPH• radical scavenging activity. Molecules.

[12] Amidžić Klarić D., Mornar A., Kovačić J., Jeličić M.-L., Brusač E., Brletić I., Klarić I. (2022). Polyphenol content and antioxidant activity of phytoestrogen containing food and dietary supplements: DPPH free radical scavenging activity by HPLC. Acta Pharm..

[13] Franzoni F., Quiñones-Galvan A., Regoli F., Ferrannini E., Galetta F. (2003). A comparative study of the in vitro antioxidant activity of statins. Int. J. Cardiol..

[14] Umeda R., Takanari H., Ogata K., Matsumoto S., Kitano T., Ono K., Tokumaru O. (2019). Direct free radical scavenging effects of watersoluble HMGCoA reductase inhibitors. J. Clin. Biochem. Nutr..

[15] Kalpana T., Karunakar N., Reddy M.S., Prabhakar M.C., Krishna D.R. (2001). Assessment of antioxidant activity of some antileprotic drugs. Arzneimittel-Forschung/Drug Res..

[16] Bhardwaj P., Garg P.K., Maulik S.K., Saraya A., Tandon R.K., Acharya S.K. (2009). A Randomized controlled trial of antioxidant supplementation for pain relief in patients with chronic pancreatitis. Gastroenterology.

[17] Kim H.K., Park S.K., Zhou J.L., Taglialatela G., Chung K., Coggeshall R.E., Chung J.M. (2004). Reactive oxygen species (ROS) play an important role in a rat model of neuropathic pain. Pain.

[18] Borges R.S., Barros T.G., Pereira G.A.N., Batista J., Beleza Filho R.F.G.P., Veiga A.A.S., Hamoy M., Mello V.J., da Silva A.B.F., Barros C.A.L. (2014). A Structure and antioxidant activity study of paracetamol and salicylic acid. Pharmacol. Pharm..

[19] Rafael J.A., Jabor J.R., Casagrande R., Georgetti S.R., Borin M.D.F., Fonseca M.J.V. (2007). Validation of HPLC, DPPH• and nitrosation methods for mesalamine determination in pharmaceutical dosage forms. Rev. Bras. Cienc. Farm. J. Pharm. Sci..

[20] Dinis C.P., Maderia V.M., Almeida L.M. (1994). Action of phenolic derivatives (acetaminophen, salicylate, and 5-aminosalicylate) as inhibitors of membrane lipid peroxidation and as peroxyl radical scavengers. Arch. Biochem. Biophys..

[21] Selva P., Srinivasan V. (2016). Antioxidant activities of ropinirole and pramipexole novel drugs used in treatment of parkinsonism: An in vitro approach. Asian J. Pharm. Clin. Res..

[22] Sigurnjak Bureš M., Ukić Š., Cvetnić M., Prevarić V., Markić M., Rogošić M., Kušić H., Bolanča T. (2021). Toxicity of binary mixtures of pesticides and pharmaceuticals toward Vibrio fischeri: Assessment by quantitative structure-activity relationships. Environ. Pollut..

[23] Rastija V., Molnar M., Siladi T., Masand V.H. (2018). QSAR analysis for antioxidant activity of dipicolinic acid derivatives. Comb. Chem. High Throughput Screen..

[24] Gramatica P., Chirico N., Papa E., Cassani S., Kovarich S. (2013). QSARINS: A new software for the development, analysis, and validation of QSAR MLR models. J. Comput. Chem..

[25] Todeschini R., Consonni V. (2008). Descriptors from molecular geometry. Handb. Chemoinform..

[26] Van Trang N., Van Trang N., Son N.T. (2020). Antioxidation of 2-phenylbenzofuran derivatives: Structural-electronic effects and mechanisms. RSC Adv..

[27] Om A.S., Kim J.H. (2007). An Approach to QSAR modeling on the radical scavenging activity of flavonoid compounds. Cancer Prev. Res..

[28] Hajimahdi Z., Safizadeh F., Zarghi A. (2016). QSAR analysis for some 1, 2-benzisothiazol-3-one derivatives as caspase-3 inhibitors by stepwise mlr method. Iran. J. Pharm. Res..

[29] Lötsch J., Ultsch A., Hähner A., Willgeroth V., Bensafi M., Zaliani A., Hummel T. (2021). Data-science based analysis of perceptual spaces of odors in olfactory loss. Sci. Rep..

